# Challenges in Diagnostic of the Ulcerative Rectitis by Lymphogranuloma venereum in Chlamydia trachomatis Infection and AIDS

**DOI:** 10.7759/cureus.35420

**Published:** 2023-02-24

**Authors:** Ana Caroline dos Santos-Ferreira, Richard Calanca, José C Ardengh

**Affiliations:** 1 Infectious Diseases, Instituto de Infectologia Emílio Ribas, São Paulo, BRA; 2 Gastroenterology, Instituto de Infectologia Emílio Ribas, São Paulo, BRA; 3 Gastrointestinal Endoscopy Department, Hospital das Clínicas de Ribeirão Preto, Ribeirão Preto, BRA; 4 Imaging Diagnosis Department, Federal University of São Paulo, São Paulo, BRA

**Keywords:** reverse transcription polymerase chain reaction (rt-pcr), hiv aids, ulcerative proctitis, ulcerative rectitis, ulcerative colıtıs, lymphogranuloma venereum, chlamydia screening

## Abstract

*Lymphogranuloma venereum* is a rare manifestation of *Chlamydia trachomatis* infection and may manifest with anorectal symptoms, rectitis, proctitis, and inguinal masses. The new outbreaks of Chlamydia infection have allowed the description of new cases with rectal symptoms (rectitis/proctitis), mainly in people living with HIV and men who have sex with men. The authors present the clinical findings in people living with HIV men who have sex with men with lymphogranuloma venereum with ulcerative rectitis identified by colonoscopy. Differentiation of proctitis was made from other causes of sexually transmitted infections, such as gonorrhea and herpes virus, inflammatory diseases (Crohn's disease), and neoplastic and opportunistic infections such as cytomegalovirus, tuberculosis, and histoplasmosis. The symptoms of the patient and the endoscopic lesions were suspected of lymphogranuloma venereum with ulcerative proctitis, which was confirmed by biopsy and performing the polymerase chain reaction. After appropriate treatment with doxycycline, the patient evolved favorably.

## Introduction

Chlamydia is a sexually-transmitted infection (STI) caused by Chlamydia trachomatis and has an extensive clinical spectrum. The Lymphogranuloma venereum (LGV) is a rare manifestation of Chlamydia trachomatis, and it is caused by specific serovars L1, L2, or L3 with Inguinal lymphadenopathy, rectitis, and proctitis symptoms [1,2]. This disease frequently occurs in young women, people living with HIV (PLHIV), and men who have sex with other men (MSM) with recent anal sexual exposure [2,3]. In recent years, outbreaks of cases with increased incidence have been described, mainly in Europe [2,3] and the USA [4]. Studies showed an increase of 75% in cases in the period 2015-2019. In 2019, in Europe, more than 3,000 cases of LGV were reported. France, Netherlands, Spain, and the United Kingdom accounted for most of them, 87% of all reported cases, and 64% were HIV positive [5]. However, as no specific molecular diagnostic tests are available, identifying these cases is complex and favors underdiagnosis.

The authors described the clinical and endoscopic findings of a case of a PLHIV and MSM with anal bleeding associated with ulcerative rectitis by LGV associated with Chlamydia trachomatis infection. The investigation required computerized tomography of the abdomen and pelvis. Colonoscopy provides the extent and involvement of the rectum and colon by the lesions, which are part of the differential diagnosis with other ulcerated digestive system forms (DS).

## Case presentation

**Figure 1:**
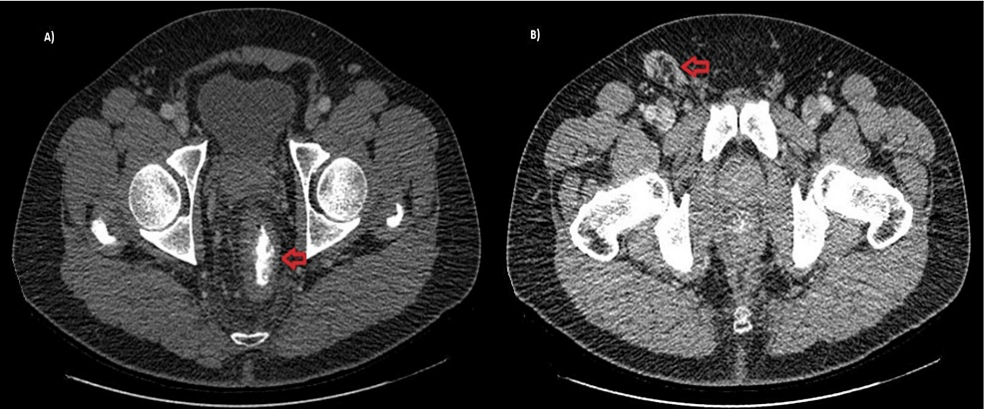
CT abdomen and pelvis with contrast in a transversal cut in portal phase phase showing in A) asymmetric parietal thickening of the distal rectum (red arrow) and in B) inguinal region with nonspecific inflammatory/infectious right inguinal lymph node enlargement (red arrow).

A male, 51 y-old, PLHIV whom MSM on the regular treatment of HIV was made with the combination of tenofovir, lamivudine, and dolutegravir, which is a highly active antiretroviral therapy (HAART), and the patient was indetectable for many years. This patient was HIV a long time ago using multiple treatments previously, and nowadays, CD4 180 cel/mm³ count could be a natural evolution of the disease. The patient presented a rectal bleed characterized by live blood and few quantities, rectal pain, and fever for 15 days, progressing with abdominal swelling and right inguinal lymph node. In the past medical history, an advanced HIV infection and histoplasmosis with anal and intestinal lesions were treated years ago. Rectal clinical examination revealed a painful anal edge Ulcer measuring 4 cm long. In the absence of genitourinary symptoms for STIs and the presence of systemic involvement in addition to risk factors for infectious and neoplastic diseases, the strategy was to perform complementary tests to define the etiology of the disease. The abdomen and pelvis CT with contrast demonstrated irregular parietal thickening of the distal rectum and right inguinal lymph node enlargement measuring 2.8 x 3.5 cm, suggestive of an inflammatory/infectious process (Figure 1).

**Figure 2:**
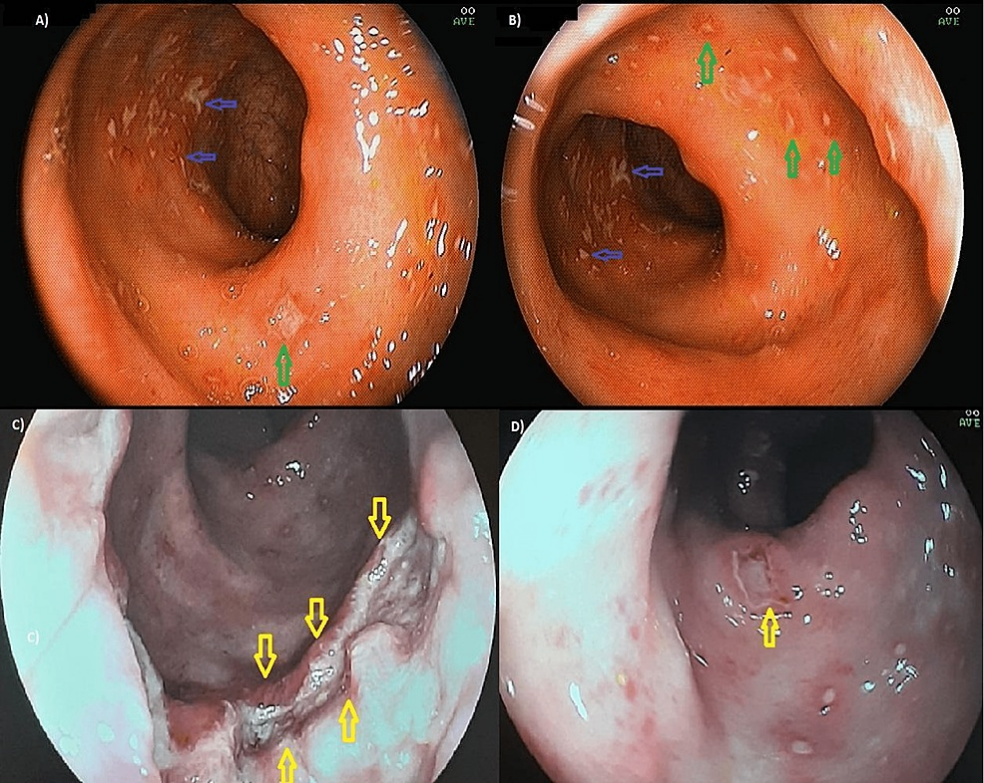
Colonoscopy. (A and B) all the entire rectum with numerous oral and serpiginous ulcerated lesions, with thick fibrin (blue arrows) and an intense halo of hyperemia around it (green arrows) between 0 and 1 cm. (C e D) Extensive, deep, and confluent ulcers with an irregular center and regular borders with star-shaped cicatricial retractions (yellow arrows).

Colonoscopy showed numerous oral and serpiginous ulcerated lesions with thick fibrin and an intense halo of hyperemia around 0.5 to 1.0 cm in the middle and distal rectum, with yellowish-white secretion on the surface and some confluence. Some lesions have irregular centers and regular hyperemic edges with stellate cicatricial retractions, with central fibrin and yellowish-white secretion and perilesional edema. The distal rectum region had an extensive and deep ulcer, a necrotic and irregular background, and a border delimited by precise limits. This aspect suggested cytomegalovirus and LGV (Figure 2).

**Figure 3:**
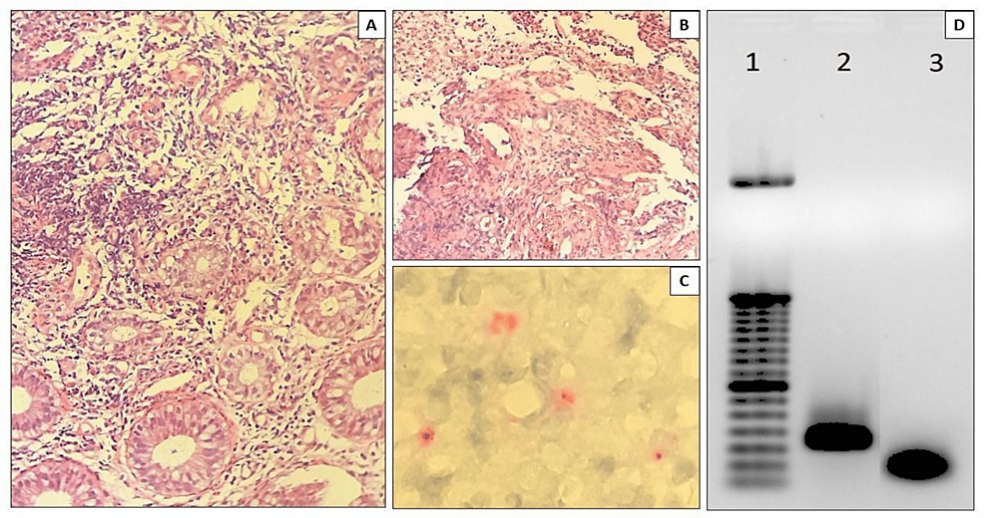
Pathological findings of the lesion: A. Intense chronic rectitis with erosive foci (HE 200X). B. Ulcer of rectal mucosa covered with fibrin-leukocyte plug, necrosis, granulation tissue, and fibrosis (HE 200X). C. Positive immunostaining for Chlamydia sp in rectal Ulcer (Immunoalkaline phosphatase with monoclonal antibody LPS strain LGV1 for Chlamydia with fast red substrate and hematoxylin counterstain 400X). D. Agarose gel electrophoresis (2%) of 23S rRNA gene fragments from Chlamydia spp. Amplified from formalin-fixed, paraffin-embedded (FFPE) anorectal ulcer tissue. Lane 1, ladder (50 bp); lane 2, positive sample recognized by the appearance of the specific band at position 130 bp; lane 3, no template control (NTC).

The histopathologic routine has slides stained with hematoxylin and eosin and showed chronic rectitis with extensive erosion, granulation tissue, and a fibrin-leukocyte plug consistent with a nonspecific ulcerated lesion (Figure 3A, 3B). Immunohistochemistry for chlamydia antigens in rectal tissue performed was positive. After deparaffinization and heat-induced epitope retrieval (citrate buffer ten mM/pH6), slides were incubated with a primary antibody, a mouse monoclonal antibody LPS strain LGV1 for Chlamydia (Chemicon MAB 8321), optimized at a dilution of 1: 500 with incubation for 18 h at 4^o^C, previously validated with negative and positive controls. The amplification signal was achieved by alkaline phosphatase-conjugated polymer (Polink-2 AP Broad Detection System; GBI Labs, WA, USA, cat. D24-110), revealed by Fast Red chromogen (GBI Permanent Red Kit; GBI Labs, WA, USA, cat. C13-120). Finally, the slides were counterstained with Mayer's hematoxylin (Polysciences, Inc.; PA, USA) (Figure 3C). Antigens (Cytomegalovirus and Herpes simplex virus) were negative. To confirm the etiological diagnosis, Formalin-Fixed Paraffin-Embedded (FFPE) anorectal ulcer biopsy tissue was cut into two serial sections with a thickness of 9 mm, placed in a microtube, and underwent DNA extraction by ReliaPrep FFPE gDNA Miniprep System (Promega; #A2351) according to the manufacturer's instructions. The samples were air-dried for 5 min at room temperature. The pellet was re-suspended in 30 uL of elution buffer, which was also used as a negative control (no template control; NTC) sample. For detecting Chlamydia spp., the PCR reaction mixture consisted of 300 nM of each primer: F 5'- GAAAAGAACCCTTGTTAAGGGAG-3'; R 5’-TTAACTCCCTGGCTCATCATG -3'5. PCR was performed following the GoTaq Green Master Mix (Promega; #M712) protocol. Cycling conditions were 95 o C for 10 min, followed by 45 cycles at 95^o^C, 56^o^C, and 72^o^C for 1 min, and one final cycle of 72^o^C for 7 min. After amplification, visualization of the 130-bp fragments of the 23S rRNA gene5 was accomplished using 2% agarose gel electrophoresis and a fluorescent nucleic acid dye (Figure 3D). These findings allowed the diagnosis of LGV, and the treatment with doxycycline was instituted for 21 days. The patient has clinical improvement.

## Discussion

This case report reveals the day-to-day diagnostic challenges for endoscopists with STIs. Anorectal ulcers are part of the syndromic diagnosis. If the immunological status of PLHIV is unknown, the possibility of opportunistic infection cannot be excluded. This fact prevents the proper treatment of the patient since the detection of the etiology may or not be associated with HIV. In this case, the normal laboratory evaluation, CT with nonspecific alterations in the rectum with inflammatory lymph node enlargement, and colonoscopy imaging alone with multiple rectal ulcers made the etiological diagnosis difficult. However, the colonoscopy with biopsy associated with immunostaining determined the definitive diagnosis.

Clinical and imaging exams led us to the syndromic diagnosis in the digestive system. The suspicion of STI cannot be ruled out due to the clinical and risk factors, and a history of anal sexual exposure is essential to be suspicious of STIs. The CD4 cells count major of 100 cells rule out the possibility of opportunistic infections, which is more common in difficult immunological situations. Another differential diagnosis of anorectal ulcers is neoplasia disease because, in PLHIV, it can occur earlier [6]. So, CT, as well as a colonoscopy with biopsy, are essential for diagnosis.

LGV infection in the primary stage may go undetected when only a painless papule, pustule, or ulceration is present. The second stage begins within 2-6 weeks after the onset of the primary lesion. It can cause an inguinal syndrome with painful inflammation and inguinal or anorectal syndrome (usually after the primary lesion of the posterior vulva, vagina, or anus). The tertiary stage is characterized by a chronic inflammatory response and tissue destruction, forming perirectal abscesses, fistulas, strictures, and rectum stenosis, a rare manifestation [7]. In LGV, infection often presents as proctitis or proctocolitis, with rectal pain, mucoid or hemorrhagic discharge, fever or tenesmus, and inguinal lymphadenopathy suppurated called "buboes' [1,4]. However, most patients with rectitis or proctitis have rectal symptoms that can also be found in infections due to gonorrhea, herpes virus, and Mycoplasma genitalium [8], so specific molecular tests are necessary to differentiate it. Chlamydia is identified based on serology (OmpA) or molecular methods [9], and particular serovars determine the clinical manifestations. LGV is caused by serovars L1, L2, and L3 and needs to be identified. These tests, meantime, are not available, and the results often need to be returned in time for the initiation of treatment [1,4].

The clinical diagnosis, based on clinical findings and risk factors associated, without microbiological exam or PCR lead us to the necessity of presumptive treatment for other STI concomitants (gonorrhea and herpes). This increases the risk of antimicrobial resistance, another emerging concern by WHO, as described in Neisseria gonorrhea disease [2,10,11]. In addition, it leads us to the underdiagnosis of LGV, which was shown in European studies [1,12]. Furthermore, the treatment of extragenital chlamydia - LGV is longer with doxycycline 100mg twice a day for 21 days and not just 7 days as in other clinical forms, which increases the risk of therapeutic failure [13].

Colonoscopy made it possible to characterize the lesions and the extent of the involvement by infection LGV, as described in other reports. Some anorectal lesions findings presented aspects suggestive of cytomegalovirus (CMV), which could not be ruled this differential diagnosis; the biopsy was necessary [13,14]. CMV ulcers can be characterized by being extensive and deep, with borders delimited in the anorectal region. In the colon [15], CMV lesions can also be aphthous with fibrin, a yellow center and well-defined borders, and an intense hyperemia halo around the ulcers, with a diffuse distribution. The literature describes LGV mimicking neoplasia [16]and inflammatory bowel disease [17] due to severe inflammation with the formation of granulomas and mucopurulent exudates. In the case presented, however, the clinical history associated with endoscopic and anatomopathological findings did not suggest these diseases. The endoscopist's experience in infectious lesions and findings can help, favoring the histological and anatomopathological evaluation by directing the line of research.

## Conclusions

The authors conclude that the case reported shows the emerging risk of LGV and the necessity of the high index of suspicion, especially in populations at risk and the risk of underdiagnosis. In front of the challenges to diagnosis, LGV treatment in the absence of specific molecular testing, as reported in European countries, should be considered. The importance of colonoscopy in the diagnosis allows for characterizing the lesions and their extension, the possible relevant differential diagnoses, and collecting tissue samples for anatomopathological and immunohistochemistry with specific molecular tests, allowing targeted treatment. The reported patient underwent specific treatment and evolved with clinical improvement of the lesions.
